# Supplementary material for: The Risk Factors and Screening Uptake for Prostate Cancer: A Scoping Review
Source: Healthcare (Basel). 2023 Oct 20;11(20):2780. doi: 10.3390/healthcare11202780 (PMC10606491; doi:10.3390/healthcare11202780)
Supplement: Supplementary file 1 [file healthcare-11-02780-s001.zip › supplementary Table S1.pdf]

Table S1: Data extraction

Author/s, Year, Title, Country	Aim or focus	Methods and methodologies	Summary of findings	Recommendation made	Limitation
[74] Adler et al. (2019) Usual adult occupation and risk for prostate cancer in West African men: the Ghana Prostate Study.<br><br>Ghana.	To examine the correlation between occupation and prostate cancer among a population with low prostate specific antigen screening rates using the Ghana prostate study.	Quantitative case control study.<br>Sample size- 1,713 participants (n=749 prostates cancer cases, n=964 controls).<br>Data collection- questionnaires.<br>Data analysis- statistical analysis.<br>Ethical approval- obtained.	Many occupations (managers and military) are exposed prostate cancer especially due to their exposure to chemicals (pesticides, fuels, solvents, radiation or shift-work chemical/warfare, metals) and sedentary lifestyle (smoking, drinking). However, there was no differences by birth, region, ethnicity among those employed in management occupations and successive prostate cancer risk.	Studies recommend a need to recognize conceivable exposures driving pragmatic increased risks.	Small number of cases and controls limiting power in some groups.
[66] Aiken and Eldemire-Shearer (2012) Prostate cancer in Jamaica and the wider Caribbean: it is time to consider screening.<br><br>Jamaica.	To discuss the role of screening and also argues for increased screening among the Caribbean population.	Discussion paper.	Race has proven to be a major contributing factor for prostate cancer in the Caribbean.<br>Nonetheless, older men are more likely to present with advance condition due to assess and lack of awareness.<br>The introduction of prostate specific antigen and digital rectal examination has proven to be a useful tool in diagnosing and early detection of the condition.	Research is needed regarding cultural views and expectations of manhood, poverty, ignorance, apathy, fatalism, stoicism, denial of risk, difficulty accessing preventive care, and specific issues related to prostate specific antigen and digital rectal examination.<br>Additionally, future research should be conducted to help clarify the significance of locally	Opinion based only.

				relevant modifiable risk factors to help develop preventive strategies that may be instituted in both the population and individual.	
[67] Åkerstedt et al. (2017) Night work and prostate cancer in men: a Swedish prospective cohort study.<br><br>Sweden.	To investigate the relationship between men exposure to night work and their risk for prostate cancer development.	Quantitative prospective cohort study.<br>Sample size- grouped 12,322 male twins.<br>Data collection- questionnaire.<br>Data analysis– statistical analysis and thematic analysis<br>Ethic approval- obtained.	The results of the study reported that, the cumulative incidence among the group who has been exposed to night work was 3.3% and that of the unexposed group was 3.9%.<br>Night workers had greater use of tobacco due to age and this may corelate to their dietary issues leading to overweight and prostate cancer risk.	The author recommends that, further research into the subject the adapt more rigorous methods that employ objective data collection methods, measures the length of exposure, and specify occupation or work tasks, is needed.	Unavailability of data on the type of work and the length of exposure to night work.
[82] Akinremi et al. (2014) Need for and relevance for prostate cancer screening in Nigeria.<br><br>Nigeria.	To investigate the acceptance for prostate cancer screening among Nigerian men and to also analyse the importance of results of screening among some Nigerian men 2005 to 2008.	Quantitative clinical investigation study.<br>Sample size- 1,124 men.<br>Data collection- blood samples and digital rectal examinations.<br>Data analysis- clinical and statistical analysis.<br>Ethical approval- obtained.	Even though prostate specific antigen screening and digital rectal examination has been agreed as the best tools for screening, knowledge and access is limited.<br>Nigerian men are willing group to undergo prostate specific antigen and digital rectal examination screening.	It is recommended that continues education, information and screening programs should be encouraged to help improve assess and awareness about prostate cancer.	A language barrier may have existed.
[33] Al Shareef et al. (2023) Risks of prostate cancer and mortality in the city of Sharjah, United Arab Emirates.	To identify risk factors for prostate cancer and death in a cohort of people who were diagnosed with the	Quantitative retrospective study.<br>Sample size- 192 cases (men 65-year+).	Study reveals 67-year-old men in Sharjah have higher prostate cancer incidence rates due to underdiagnosis, genetic	Further studies are needed to confirm findings and identify risk factors and protective	Study's retrospective design may introduce selection biases.

United Arab Emirates.	disease between 2012 and 2021 in Sharjah, United Arab Emirates.	Data collection- questionnaire.<br>Data analysis- statistical analysis.<br>Ethical approval- obtained	predisposition, environmental factors, and infection.<br>Prostate specific antigen density is a potential prostate cancer screening marker, potentially reducing unnecessary biopsies in populations with high benign prostatic hyperplasia prevalence.	factors affecting prostate cancer outcomes.	
[70] Alkhatib et al. (2022) Does veteran status mitigate racial disparities in prostate cancer screening: Analysis of prostate specific antigen screening patterns in the 2018 behavioral risk factor surveillance system data.<br><br>United states of America.	To evaluate how veteran status affected prostate-specific antigen screening and if veterans' access to health insurance helps to reduce racial inequalities in screening.	Quantitative- cross-sectional study.<br>Sample size- 21,517 veterans (non-Hispanic White and non-Hispanic Black men).<br>Data collection- questioner<br>Data analysis- statistical analysis.<br>Ethical approval- obtained.	A 40% prostate specific antigen screening prevalence.<br>Veterans had higher rates that nonveterans.<br>A significant interaction was found between veteran status and race, with Non-Hispanic Black veterans having higher odds of undergoing prostate specific antigen screening compared to nonveterans. This association was not demonstrated when comparing Non-Hispanic White veterans versus nonveterans.	Additional research is required to apply these findings.	Study's retrospective design may introduce selection biases.
[85] Austin (2012) Prostate-specific antigen prostate cancer screening: Answers to the critical questions.<br><br>United States of America.	To update long-term care providers about the significance for prostate cancer screening regarding the current randomized trial.	Discussion paper.	Although prostate-specific antigen is useful in the prostate cancer screening, it also a controversial issue which affect long term care.<br>Due to this, most clinicians do not involve	Clinician should involve their patient in decision making before initiating prostate specific antigen screening.	Expert opinion or non-researched based paper.

			their patient in decision making before they commence screening. The prostate specific antigen level is recognised as ≥4.0 to 4.5 ng/ml. Continues use of prostate specific antigen leads to overdiagnosis and may bring harms exceeding benefits.		
[34] Baade et al. (2005) Communicating prostate cancer risk: What should we be telling our patients.<br><br>Australia.	To discuss communications on prostate cancer risk: what should health professionals should be telling their patients.	Discussion paper.	The cause for prostate cancer is unknown but predisposing factors such as family history, age, diet, exposure to environmental hazards and race (African American men) can lead to prostate cancer. Population-based screening is not recommended, but screening should be allowed if men are well informed about the pros and cons of screening. Decision making should be made by patients who understand their vulnerability to screening.	Men need to be supported and encouraged to go for screening due to their vulnerability.	Non-research based paper expert opinion only.
[35] Beaulac et al. (2006) Lifetime and recent prostate specific antigen (PSA) screening of men for prostate cancer in Canada.	To inform people on prostate screening by determining the occurrence of prostate specific antigen	Discussion paper.	Age, ethnicity, family history is some of the known nonmodifiable risk factors for prostate cancer.	Future studies should focus why prostate specific antigen screening is not congruent with national screening	Expert opinion Paper. Data is self-reported.

Canada.	screening and test, and socio demographic characteristics in Canada.		Almost half of Canadian men reported a lifetime prostatic specific antigen screening event.<br>However, prostate specific antigen screening was related to those who earn higher income.<br>Due to language barrier, most people are unlikely to get prostate screening.<br>Smokers had a higher rate of getting screened while men of black ethnicity were not likely to be screened.	guideline to increase the access.	
[36] Berenguer et al. (2023) Underlying features of prostate cancer-statistics, risk factors, and emerging methods for its diagnosis.<br><br>Portugal.	To summarize prostate cancer data including incidence and mortality rates, risk factors and screening techniques.	Expert opinion paper.	Prostate cancer risk factors include advancing age, ethnicity, genetic mutations, insulin-like growth factors, family history, and lifestyle.<br>African American, Caribbean, and black men have the highest incidence, with complex genetics.<br>Lifestyle factors like diet, exercise, and environment significantly reduce cancer risk, with 30-50% of cancers prevented through healthy choices, avoiding tobacco, alcohol, and public health measures.<br>Screening for prostate cancer involves prostate	To address digital rectal examination some of the specificity and sensitivity issues with present screening methods, advancements in prostate cancer diagnoses and therapy are needed.	Expert opinion non-research based paper.

			specific antigen biomarker values in blood serum and digital rectal examination.<br>After suspicion, magnetic resonance imaging scans determine biopsy options.<br>Staging tests, such as cat scan or positron emission tomography, may reveal benign tumours.<br>Prostate specific antigen screening for prostate cancer has limitations in selectivity, sensitivity, accuracy, and specificity, and can be influenced by factors like age, body mass index and infection.		
[37] Bergengren et al. (2023) 2022 update on prostate cancer epidemiology and risk factors: A systematic review.<br><br>United States of America.	To examine and synthesize the most recent data on descriptive epidemiology, extensive screening trials, diagnostic approaches, and prostate cancer risk factors.	Systematic review.	Age, family history, genetic predisposition, height, physical activity, sleep, diet, and macronutrients are significant risk factors for prostate cancer.<br>Height is linked to increased risk, possibly due to insulin-like growth factor levels during puberty.<br>Physical activity and sleep have no clear increased risk, while diet has a weak protective association.<br>Alcohol and smoking increase prostate cancer	Future systematic review should focus on the use biomarkers for screening prostate cancer.	Meta-analyses may not include clinical prostate subtypes.<br>Review does not examine prostate specific antigen screening in studies.

			risk, while infertility and vasectomy vary. Secondary prevention and early detection are gaining acceptance, with guidelines recommending organized screening programs using biomarkers.		
[71] Blanchard et al. (2005) Knowledge, attitudes and beliefs of women about the importance for prostate cancer screening.<br><br>United States of America.	To evaluate and understand the knowledge, beliefs and attitude of women regarding prostate cancer and further educate the men on the importance of screening and early detection.	Quantitative cross-sectional study.<br>Sample size- 324 women.<br>Data collection- questionnaire.<br>Data analysis- statistical analysis.<br>Ethical approval- obtained.	Race (African American men) are at the highest of been diagnosed for prostate cancer.<br>Prostate specific antigen and digital rectal examination have been recognised as tools for prostate cancer screening.<br>Most people feel that screening with prostate specific antigen and digital rectal examination makes them feel uncomfortable.	Future similar studies utilising other ethnicities are recommended to develop a good strategy for education.<br>Need to evaluate whether the application of a family-based education program will relate knowledge for prostate cancer and participation in screening and early detection by men.	Limited to one specific race.
[86] Bowen (2011) Prostate cancer screening and informed decision-making: provider and patient perspectives.<br><br>United States of America.	To determine the level of informed decision making for prostate cancer screening in a definite population.	Mixed method.<br>1 - Sampling- data from the Washington State<br>Data collection- interview.<br>Data analysis- qualitative analysis.<br>2- Sampling- 700 primary care physicians.<br>Data collection- questionnaire.<br>Data analysis – statistical analysis.<br>Ethical approval- obtained.	Using prostate specific antigen screening for prostate cancer seems accepted by the participants.<br>However, most health practitioners do not used decision making aids to assist their patient during the process rustling in high rate of screening in men.	Health professionals must engage in framing prostate cancer screening as a decision and not a conclusion.<br>There should be a national policy that will guide health professional to guide patient make an informed decision not forced into screening.	Providers sampled may not match patients surveyed, affecting prostate cancer screening role.

[38] Brawley et al. (2009) Screening for prostate cancer.<br><br>United States of America.	To discuss the screening for prostate cancer.	Discussion paper.	Continuous screening leads to the identification of unknown tumours leading to overdiagnosis and treatment.<br>Prostate specific antigen and digital rectal examination is recognised as essential tool for screening with the prostate specific antigen threshold of 4.0 – 4. 5 ng/ML.<br>Risk factors such as family history, race/ethnicity, and age are considered before prostate biopsy becomes an option.	Future studies should focus on how physicians should engage patients in shared decision-making for prostate cancer screening, considering recent United States and European studies' outcomes.	Only expert opinion.
[39] Calabrese (2004) Prostate cancer in older men.<br><br>United States of America.	To discuss and identify an overview of prostate cancer, treatment options, and side effects in relation to older men.	Discussion paper.	There are several causes for prostate cancer in older men, and include increasing age (65+), race, family history, and dietary intake (fats).<br>Generally screening for prostate cancer is seen as complex.<br>The use of prostate specific antigen test, digital rectal examination, and biopsy have been accepted to be effective.	Continues research is recommended to help identify the best way for screening older men.<br>Health professionals need to educate, support, and listen to the patients.	Non-research based paper expert opinion only.
[87] Carlsson and Vickers (2020) Screening for prostate cancer.	To discuss prostate cancer screening.	Discussion paper.	Prostate cancer screening has evolved due to prostate-specific antigen testing but it may cause undesirable anxiety or	Shared decision making should be enhanced to reduce any stress or anxiety.	Not a researched based paper.

United States of America.			stress due to false negative results and complications.<br>Prostate specific antigen screening can lead to overdiagnosis and treatment and as a result patient can refuse screening.<br>Patient should be involved in decision making about screening.		
[88] Carter (2006) Assessing risk: Does this patient have prostate cancer.<br><br>United States of America.	To comments on a publication by Thompson et al. 2006 on prostate-specific antigen levels and their use in risk assessment and diagnosis for prostate cancer.	Editorial.	In assessing risk factors and diagnosing prostate cancer based on threshold of prostate specific antigen can cause overdiagnosis and overtreatment of the disease.<br>Most men who were diagnosed for prostate cancer has prostate specific antigen levels below 4.0ng/ml.<br>Therefore, no specific prostate specific antigen threshold can be recommended to rule out prostate cancer.	In the absence of accurate markers for prostate cancer other approaches to predicting the risk such as family history, digital rectal exam and biopsy should be incorporated to increase the diagnosis for prostate cancer.	This is an editorial and information based primarily on one study.
[89] Caso (2010) Prostate cancer: an evolving paradigm.<br><br>United States of America.	To discuss prostate cancer as an evolving paradigm.	Expert opinion.	The use of prostate specific antigen for screening has brought about overdiagnosis and overtreatment.<br>However, rates of prostate specific antigen recurrence have also decreased significantly	Future research in tumour biology of early-stage prostate cancer is needed to help reduce treatment before active confirmed diagnosis.	Expert opinion only.

			due to other screening like the use of biopsy and digital rectal examination		
[40] Castillejos-Molina and Gabilondo-Navarro (2016) Prostate cancer.<br><br>Mexico.	To discuss screening diagnostic methods, and treatment for prostate cancer patients.	Expert opinion.	Age and hereditary are seen as the highest cause of risk factors for prostate cancer.<br>Hormones, race, and some fatty foods are being recognized to caused prostate cancer.<br>An effective way is the detection of high levels of prostate specific antigen levels and palpating with digital rectal exam.<br>Prostate biopsy is an effective way for screening and diagnosing prostate cancer.	Involving the patient throughout screening with or without the use of prostate specific antigen and digital rectal examination.<br>Encourage regular exercise to reduce the risk for prostate cancer.	Expert opinion non-researched based paper.
[41] Chang (2023) A matched case-control study in Taiwan to evaluate potential risk factors for prostate cancer.<br><br>Taiwan.	To identifies prostate cancer risk factors, including human papillomavirus infection detection, dietary, family, lifestyle, and sexual behaviour.	Quantitative matched case control study.<br>Sample size- case group 143 and control group 135 patients.<br>Data collection- questionnaire.<br>Data analysis- statistical analysis.<br>Ethical approval- obtained.	No significant differences in age, height, weight, body mass index between prostate cancer and benign prostate hypertrophy groups.<br>Prostate cancer patients had higher occupational exposure and human papillomavirus positivity.<br>Positive correlation between prostate cancer and family history, circumcision reduces risk, vasectomy increases long-term risk.<br>Prostate cancer patients had more lifetime	Although there has been a lot of attention paid to the link between human papillomavirus and prostate cancer the findings of this study suggest that further work should be done to investigate dietary practices, individual lifestyles, and family history as potential prostate cancer risk factors.	Variations in results dues to geographical and ethic difference.

			partners, while benign prostatic hyperplasia patients have fewer partners.		
[42] Chen et al. (2014) Prostate cancer in Asia: a collaborative report.<br><br>China.	To summarize the latest epidemiology information, risk factors, racial differences, new trends in surgery management and novel agents for castration-resistant in prostate cancer.	Expert opinion.	The causes for prostate cancer in Asian men can be caused by genetics, however, mostly low in Asian men.<br>Prostate cancer detection with prostate specific antigen differs in the different kinds of Asian countries	Continuous research will help Asian researchers to understand and identified the trends for prostate cancer in their country.	Expert opinion.
[43] Childre et al. (1998) Prostate cancer: Clinical perspectives.<br><br>United States of America.	To discuss current diagnostic and therapeutic options available providing insight into the occupational care professional's role in detecting and treating prostate cancer.	Discussion paper.	Cause for prostate cancer remains unclear, but factors such as environmental, genetics, race, age, diet (fatty diet) contribute.<br>Frequency of sexual activity and vasectomy do not cause prostate cancer.<br>Prostatic specific antigen and digital rectal examination together have been demonstrated to detect almost 100% of prostatic malignancies.	The use of videos and pamphlets to educate occupational health professionals to increase their knowledge on screening is suggested to be effective.	Opinion paper only.
[44] Chung et al. (2019) The incidence, mortality, and risk factors for prostate cancer in Asian men.<br><br>South Korea.	To define the risk factors and epidemiology for prostate cancer in Asian men and compare it to western data in the past 10 years.	Systematic review.	Asians in Western countries are at a higher risk of prostate cancer than those in the Asians countries which may be due to lack of prostate specific antigen screening.	Eating healthy, regular exercise may turn to reduce the risk of one getting prostate cancer.	Few data bases were used and a limited timeframe.

			In Asia modifiable risk factors such as environmental exposures, dairy products and meat may enhance one getting prostate cancer.<br>In Europe and United States age and family history is seen the non-modifiable risk factors.		
[45] Clinton and Giovannucci (1998) Diet, nutrition, and prostate cancer.<br><br>United States of America.	The goal of review focuses on evidence that diet and nutrition play a role in the aetiology and prevention for prostate cancer.	Discussion paper.	Age, race, occupational exposure, family history, genetics and migrations can be some of the causes for prostate cancer.<br>Fruits and vegetables, fish, calcium rich diet and physical activity may produce some energy balances which may prevent prostate cancer, while the intake of fatty foods, sodium and meats lead to a possible cause of the disease.	There should be surety when providing empirical evidence in future research.	Inconsistencies in some of the evidence provided.
[77] Consumer Protection (2001) Best test and care for prostate cancer.<br><br>United States of America.	To highlight the best of test and care of patients with prostate cancer.	Discussion paper.	Prostatic specific antigen testing is helping to improve quality of care and save lives resulting in a decline in prostate cancer cases.<br>Different threshold has been employed by doctors in diagnosing prostate cancer and this has an impact on the age of which prostate cancer can be diagnosed.	Prostate specific antigen screening will help men (50 years) to add 10 years more in their lives.<br>Prostate specific antigen should be combined with digital rectal examination to help detect all possible case for prostate cancer.<br>The best accuracy to improve prostate specific antigen test is to lower the values for men in their 40s and 50s.	Non-research based paper.

[90] Crittendon et al. (2022) Does perceived racism affect prostate cancer screening rates and patient-provider shared discussions among black and white men.<br><br>United States of America	The purpose of this study was to look at the relationships between perceived racism, prostate screening rates and shared medical discourse.	Quantitative cross-sectional study.<br>Sample size – 14,645 men.<br>Data collection- questionnaire.<br>Data analysis- statistical analysis.<br>Ethical approval- obtained.	No significant difference in prostate specific antigen testing rates between black men and white samples, despite historically marginalized black men being hard to engage in research due to mistrust, fear, and perceived data use.<br>No significant relationship between perceived racism and prostate specific antigen outcomes, but black men face differential treatment in workplace and healthcare.	The application of critical race theory as a primary framework for future research on black men's health is crucial to address digital rectal examination historical biases, increase preventive care rates, and reduce the risk of inadvertent harm due to incomplete shared decision-making and poorer quality of care.	Study's data after 2010 limited to 641 black men, affecting representativeness.<br>Limited data on physician race's impact on medical care/communication.
[78] da Rocha Araujo et al. (2018) Prostate cancer screening: Beliefs and practices of the Brazilian physicians with different specialties.<br><br>Brazil.	To evaluate the beliefs and practices of Brazilian physicians of different specialties with regards to prostate cancer screening.	Quantitative cross-sectional study.<br>Sample size- 275 {general practitioners (96), geriatricians (85), and urologists (94)}<br>Data collection- questionnaire.<br>Data analysis- statistical analysis.<br>Ethical approval- obtained.	Although it was established that most (81%) prefer the use of prostate specific antigen and digital rectal examination for screening, some participants (6.5%) did not seek the concerns of their patient before initiating the process.<br>Majority agreed that screening should start from 40 years.	Future research should focus the role of digital rectal examination in prostate cancer with regards to the relevance of the outcome, (mortality and quality of life).	Difference in sample size, may bring about ungeneralised result Inconsistency in some responses.
[91] de Bekker-Grob et al. (2013) Men's preferences for prostate cancer screening: A discrete choice experiment.<br><br>Netherland.	To identify men's preferences for prostate cancer screening and the trade-offs they make.	Quantitative cross-sectional study<br>Sample size- 1,000 men.<br>Data collection- questionnaire.<br>Data analysis- statistical analysis.	Prostate cancer screening aspects significantly influenced men's preferences, with risk reduction, unnecessary treatment, and costs being important factors.	Further research is needed to determine physicians' and men's preferences for prostate screening.	Selection bias during sampling. Lack of understanding of the participants to make a choice during screening.

		Ethical approval- obtained.	Men's preference for prostate cancer screening, highlighting the need for tailored programs for informed decision-making.<br>Main choice digital rectal examination and prostatic antigen test.		
[46] De Silva and Alcorn (2022) A tale of two cancers: A current concise overview of breast and prostate cancer.<br><br>Canada.	To provide pertinent, condensed information about breast and prostate cancer.	Expert opinion.	Early prostate cancer may show symptoms like breast cancer, with risk factors including age, ethnicity, race, geography, family history, and genetic changes.<br>Genetic predisposition and environmental factors contribute to prostate cancer risk, with family history accounting for 20% of cases.<br>Western lifestyles may increase cancer incidence.	Further research needs to focus on only prostate cancer to help in conducting a wholesome review.	Expert opinion only non-research based paper.
[47] Drudge-Coates (2012) Prostate cancer overview Part 1: Non-metastatic disease.<br><br>United Kingdom.	To provide an epidemiological overview of risk factors, anatomy, presentation and diagnosis, alongside staging and treatment for prostate cancer and its impact in non-metastatic disease.	Expert opinion.	The risk for prostate cancer can strongly be related to age (50+) and rarely seen in men under 40 years.<br>Family history, race, diet and genetics are risk factors for prostate cancer.<br>Prostatic specific antigen and digital rectal examination are tools that aid in diagnosing prostate	It is recommended that nurses should be well educated to help discuss the risk factors and screening uptake to support patient decision making.	Expert opinion non-research based paper.

			cancer and transrectal ultrasound and prostate biopsy can be used in confirmation.		
[48] Dunn and Kazer (2011) Prostate cancer overview.<br><br>United States of America.	To discuss the anatomy, risk factors and epidemiology, clinical manifestations, diagnosis, stages of progression, treatment, emerging therapies, and patient education for prostate cancer.	Discussion paper.	Advanced age is related to developing prostate cancer.<br>Race and family history are the second most common risk factors.<br>Prostate needle biopsy and transrectal ultrasound guided needle biopsy is wildly used to assess prostate tissues and screening.<br>Digital rectal examination and prostatic specific antigen test can be combined in screening to attain diagnosis.	Further research on quality-of-life issues and evidence-based symptom management.	Expert opinion or non-researched based paper.
[49] Firzara and Ng **(**2016) Knowledge and practice for prostate cancer screening among general practitioners in Malaysia: A cross-sectional study.<br><br>Malaysia.	To determine private general practitioners' knowledge and practice on prostate cancer screening.	Quantitative cross-sectional study.<br>Sample size– 311 general practitioners.<br>Data collection- questionnaire.<br>Data analysis- statistical analysis.<br>Ethical approval- obtained.	Respondents identified family history (82.7%) and increased age of more than 50 years (97.4%) as risk factors for prostate cancer.<br>49.5% would screen asymptomatic for prostate cancer and 94.9% would use prostate specific antigen.<br>Some general practitioners would not inform patients before conducting prostate specific antigen screening.	Continuous education and training of private general practitioners.<br>A need for national prostate cancer guidelines.<br>A need for a decision support tool to assist patients make an informed decision about prostate cancer screening.<br>Future qualitative studies to explore factors that influence general practitioners' decision to perform or screen for prostate cancer.	Public and rural general practitioners were excluded.

[50] Gaines (2010) Controversies in prostate cancer screening and treatment.<br><br>United States of America.	To discuss controversies in prostate cancer screening and treatment.	Expert opinion.	Prostate specific antigen screening and digital rectal examination are used for prostate screening in early detection and reducing risk of death.<br>However, based on individual factors such as age, race, family history and previous biopsy having been conducted.	A standard age limit should be set to help in early detection and reduce reoccurrence of the condition.	Expert opinion only.
[51] Gontijo Gomes et al. (2015) Risk factors for prostate cancer, and motivational and hindering aspects in conducting preventive practices.<br><br>Brazil.	To recognise the risk factors for prostate cancer, its preventive practices, barriers and motivating factors for disease.	Quantitative cross-sectional study.<br>Sample size- 92 males.<br>Data collection- questionnaire<br>Data analysis- statistical analysis.<br>Ethical approval- obtained.	Majority of participants (70.7%) aged over 50.<br>Age a major risk factor for prostate cancer with both incidence and mortality rates increase significantly.<br>19.6% indicated that ingesting a diet high in saturated fat will lead to prostate cancer.<br>18.5% had family history for prostate cancer.<br>Few participants felt motivated towards prostate specific antigen and digital rectal examination screening due to lack of access, fear and distrust.	Conducting early education among young people about any signs or symptoms of prostatic change will help to increase awareness and encourage them to maintain healthy lifestyle.	Non validated data collection instrument.<br>Small sample size.
[52] Gray (2002) A prostate cancer primer.<br><br>United States of America.	To familiarise nurses with the basic facts about prostate cancer and its management.	Discussion paper.	Age, race/ethnicity, family history, genetics, hormone and diet were identified as some of the predisposing factors for prostate cancer.	Further research is needed to establish that BRCA1 gene and other genetics cause an effect on prostate cancer risk.<br>Future research is needed to establish the link that	Non-research based paper expert opinion only.

			Risk of prostate cancer can vary proportionately among age or ethnicity.<br>For early detection digital rectal examination and prostatic antigen test were used.<br>Prostate biopsy and transrectal ultrasound are needed to confirm.	vasectomy can cause prostate cancer.	
[53] Harvard Health Publication (2015) Prostate cancer: What's your risk.<br><br>United States of America.	To discuss prostate cancer and explores the risk factor.	Discussion paper.	The likelihood of an American man getting prostate cancer is about 3% and this prognosis can be increased by risk factors such age, family history, and race or ethnicity.<br>The consumption of proper diet can help reduce the risk of prostate cancer.	It is recommended that eating more vegetables and less red meat and processed diet can likely reduce one's risk of developing prostate cancer.	Non-research based opinion paper.
[72] Held-Warmkessel (2002) What your patient needs to know about prostate cancer.<br><br>United States of America.	To discuss what your patient needs to know about prostate cancer.	Expert opinion.	African Americans as a race has been recognised as the people at a higher risk of getting prostate cancer.<br>Young men suspected and older people above 50 years should be screened yearly with the use of prostate specific antigen level and digital rectal examination.<br>If prostate cancer suspected transrectal ultrasound with biopsy recommended for confirmation.	Engage in screening when prostate is suspected.<br>Encourage regular screening.	Non-research based opinion paper

[54] Hoffman et al. (2022) Examination of prostate-specific antigen (PSA) screening in military and civilian men: Analysis of the 2018 behavioural risk factor surveillance system.<br><br>United States of America.	The aim of the study is to ascertain if prostate-specific antigen screening rates among military men differ from those of civilian men and whether shared decision-making Is related to prostate specific antigen screening.	Quantitative study.<br>Sample size– 101,901 men.<br>Data collection- data from the 2018 Behavioural Risk Factor Surveillance System.<br>Data analysis- statistical analysis.<br>Ethical approval- obtained	Prostate specific antigen screening is higher in military men due to increased awareness of prostate cancer due to occupational exposures.<br>Military personnel with Agent Orange exposure are twice as likely to develop prostate cancer, develop it younger, and have a more aggressive variant.<br>Military and civilian men's prostate cancer screening rates may vary due to changes in recommendations, family history, race, and age.<br>Military men have higher shared decision-making levels than civilians and are three times more likely to receive prostate specific antigen tests.	Further research is needed to understand the role of discussing advantages versus disadvantages with a provider in prostate specific antigen testing decision.<br>Further research is needed to make the shared decision-making process more robust and clinically relevant for both military and civilian populations.<br>Further research is needed to understand the military healthcare system's care processes and how universal access to care increases the likelihood of appropriate preventive care.	Limited detail provided on military service history.
[76] Ito (2014) Prostate cancer in Asian men.<br><br>Japan.	To discuss the epidemiology observations and theoretical considerations relating to the incidence, screening and mortality for prostate cancer in Asia.	Discussion paper.	Low prostate cancer in Asian and native countries due to lower exposure to prostate specific antigen screening in Asian countries.<br>The use of prostate specific antigen improved screening among the people in the region.<br>Screening methods and prostate specific antigen	The best approach should be used in screening in various Asian countries to improve future research.	Discussion paper non-research based paper.

			levels differs in the various Asian countries due to diversity.		
[75] Jochems et al. (2023) Smoking and risk of prostate cancer and prostate cancer death: A pooled study.<br><br>Sweden.	To examine prediagnosis smoking behaviour (status, intensity, duration, and cessation) as a risk factor for prostate cancer incidence and mortality, both on its own and in combination with body mass index.	Quantitative prospective cohort study.<br>Sample size– 351,448 men.<br>Data collection- five Swedish cohorts.<br>Data analysis- statistical analysis.<br>Ethical approval- obtained.	Smoking intensity, duration, and cessation are associated.<br>Combining smoking with obesity decreases low-risk prostate cancer incidence and increases prostate cancer death risk.	Future research must determine whether quitting smoking after receiving a prostate cancer diagnosis improves prognosis.	Study limited to men's smoking habit information at prostate cancer diagnosis.
[55] Johnson (2016) Prostate cancer: Can I have a prostate specific antigen test please nurse.<br><br>United Kingdom.	To discuss signs, symptoms and risk factors for prostate cancer and the evidence for and against prostate specific antigen testing.	Expert opinion.	It was recognised that advancing age (65+) is linked to prostate cancer.<br>Other risk factors include ethnicity and family history, genetics and race (African American).<br>Little agreement on screening method with prostate specific antigen screening and prostate biopsy being most accepted.	It was recommended that nurses acquire more knowledge about prostate specific antigen screen to help educate their patient in making their choice with regards to screening.	Opinion based non-research based paper.
[92] Kabore (2014) Knowledge and awareness for prostate cancer among the general public in Burkina Faso.<br><br>Burkina Faso.	To evaluate knowledge for prostate cancer among men with different levels of education in the main city of Burkina Faso (Ouagadougou).	Quantitative cross-sectional study.<br>Sample size- 600 men.<br>Data collection- questionnaire.<br>Data analysis-statistical analysis.<br>Ethical approval- obtained.	There was a deficit in knowledge about prostate cancer.<br>Prostate specific antigen blood test and digital rectal examination are tools used for screening and early detection.<br>Participants had a low knowledge of screening tools but aware of screening (70.3%).	A well-documented national prostate cancer awareness policy or plan should be introduced to enhance public education.	Sample and results did not represent all men in Burkina Faso (Ouagadougou).<br>The study only focusses on educational background neglecting other demographics.

[68] Kabore et al. (2011) Prostate cancer outcome in Burkina Faso.<br><br>Burkina Faso.	To identify the incidences for prostate cancer diagnosis characteristics in Burkina Faso.	Quantitative prospective nonrandomised cohort study.<br>Sample size- 166 patients.<br>Data collection- cases diagnosed by histological analysis of transrectal prostate biopsies.<br>Data analysis- statistical analysis.<br>Ethical approval- obtained.	Old age and race (African American) have periodically been seen as a major contributing cause for prostate cancer. While diagnostic investigation such as prostate specific antigen, digital rectal examination and transrectal prostate biopsy may show a significant way of screening and identifying the condition.	Future studies should focus or racial outcomes for prostate cancer.	Lack of information provided to the participants.
[83] Kagotho and Tan (2008) Predictors for prostate cancer screening among older immigrant men.<br><br>United States of America.	To examine the predictors for prostate cancer screening among legal permanent immigrant men aged ≥50 years holding residential status.	Quantitative cross-sectional observational study.<br>Sample size- 166 immigrant men.<br>Data collection- A modified version of the behavioural model of health services was used for vulnerable populations.<br>Data analysis- - statistical analysis.<br>Ethical approval- obtained.	Even though prostate specific antigen screening is recognised as an effective tool in prostate cancer screening, most migrants found it difficult to assess prostate cancer screening due to region of origin, access to medical care, language, age, education and visa adjustment status.	Future research is needed to extensively analyse barriers affecting immigrant men in assessing prostate specific antigen screening.	Reading and understanding (literacy issues).
[93] Kalavacherla et al. (2023) Low-value prostate-specific antigen screening in older males.<br><br>United States of America.	To identify the elements connected to low-value prostate specific antigen screening in males aged 70 or older.	Quantitative prospective cohort study.<br>Sample size-32,306 men.<br>Data collection- cases diagnosed by histological analysis of transrectal prostate biopsies.<br>Data analysis- statistical analysis.<br>Ethical approval- obtained.	Prostate specific antigen screening rates in 70+ males increased by 39.4%, driven by factors like primary care physician education, and annual income.<br>Older males with high income and education use screening more frequently, with American Indians having the lowest screening rate.	Future research should quantify the effects of this screening, particularly those of overtreating patients.	The prostate specific antigen screening module was optional, causing sampling bias. The survey did not differentiate between prostate specific antigen tests for prostate cancer screening and monitoring noncancer prostate diseases.

			Marriage had an influence on screening usage.		
[96] Kaninjing et al. (2018) Prostate cancer screening perception, beliefs, and practices among men in Bamenda, Cameroon.<br><br>Cameroon.	To scrutinize the perceptions, beliefs, and practices of Cameroonian men regarding last unite diagnoses; recognized factors that influence screening decision; and determined how men definite between traditional or predictable medicine for prostate cancer diagnosis and treatment.	Qualitative study.<br>Sample size- 25 men.<br>Data collection- focus group interviews.<br>Data analysis- qualitative data analysis.<br>Ethical approval- obtained.	The key factors that informed the decision of the men to avoid going for screening were cultural or religious beliefs, inadequate knowledge and awareness for prostate cancer and screening methods, attitude toward prostate cancer and stigma connected with the illness.	It is recommended that future studies should consider using a larger sample in conducting this study.<br>Future research should focus on prostate cancer survivors by generating ideas for designing a cancer support intervention.	Small sample size.<br>Some answers were bias because most of the participant gave information which was socially desirable.
[97] Kilpeläinen et al. (2016) Prostate cancer and socioeconomic status in the Finish randomized study of screening for prostate cancer.<br><br>Finland.	To determine if screening minimizes the difference in prostate cancer risk based on socioeconomic class.	Quantitative prospective cohort study.<br>Sample size- 72,139 men (28,678 in the screening arm and 43,461 in the control arm).<br>Data collection- Finnish Cancer Registry.<br>Data analysis – statistical analysis.<br>Ethical approval – obtained.	Men of a high socioeconomic class are more active in screening programs, but prostate cancer screening remains complex due to adverse effects.<br>Improvements in sensitivity and specificity are needed.<br>Organized screening increases low-risk prostate cancer risk by 58%-80% across socioeconomic groups, with moderate income and tertiary education being the largest difference.	To increase participation in population-based cancer screening, more focus should be paid to enrolling men from low-socioeconomic status groups.	1,493 men who were randomized to the screening arm never received an invitation and thus never participated in the screening protocol.
[80] Kim and Alhassan (2023) Analysing factors enabling prostate cancer screening behaviours	To explore the relationships between cultural distrust and African American males'	Quantitative cross-sectional study.<br>Sample size- 304 men.	The findings demonstrated that yearly family income and annual health checkups	More representative samples of African American men are required in future	The use of non-probability sample from the seminar attendees

among African American males in the south region using the Andresen's behavioural model of healthcare services utilization.<br><br>United States of America.	screening behaviors for prostate cancer in the Southeast area.	Data collection- questionnaire.<br>Data analysis– statistical analysis.<br>Ethical approval – obtained.	were significantly connected to prostate cancer screening behaviours.<br>However, the effect of health insurance, awareness, racism and past experiences were reduced among older African American males.	research to have a full understanding of their health-related screening behaviours.	making it difficult to generalise the results.
[99] Koitsalu et al. (2018) Predictors of participation in risk-based prostate cancer screening.<br><br>Sweden.	To identify important predictors of involvement in risk-based prostate cancer screening, including prostate cancer worry, knowledge of prostate cancer, health behaviour, and health-related quality of life.	Quantitative cross-sectional study.<br>Sample size- 10,000 men.<br>Data collection- questionnaire.<br>Data analysis– statistical analysis.<br>Ethical approval – obtained.	Decliners were not worried or perceived prostate cancer risk lower.<br>Higher prostate cancer knowledge predicts participation in screening.<br>70% of respondents interested in prostate cancer screening, despite lack of prostate cancer information.	Future studies should focus on what causes of worry in prostate cancer patients.	Lack of socio-demographic and medical information.
[79] Linn et al. (2007) Prostate-specific antigen screening: friend or foe.<br><br>United States of America.	To discuss the medical evidence and disagreement surrounding prostate specific antigen screening.	Discussion paper.	Screening with prostatic specific antigen and digital rectal examination and has been recognised as an important tool in diagnosing prostate cancer.<br>However, a single prostate specific antigen was seen to be more accurate than digital rectal examination.	Men over 50 should be screened with both prostate specific antigen and digital rectal examination.<br>Screening should be recommended or done annually for high-risk men and those above 45 years.	Expert opinion paper only non-research based.
[94] Maclean (2020) Probing the prostate: what everyone should	To discuss the diagnosis, prevalence and risk	Discussion paper.	Prostate-specific antigen testing has been recognized as an	Continuous education would help people	Non-research based paper expert opinion only.

know about the most commonly diagnosed cancer in Canadian men.<br><br>Canada.	factors for prostate cancer in Canada.		important tool for the diagnosis and monitoring for prostate cancer and often used in conjunction with a digital rectal exam.<br>To confirm diagnosis the gold standard prostate biopsy and biomarker tests should be used.	identify the risk factor for prostate cancer.	
[56] Maladze et al. (2023) Knowledge and attitudes towards prostate cancer and screening among males in Limpopo province.<br><br>South Africa.	To explore males' awareness, attitudes, and prostate cancer screening behaviour.	Quantitative cross-sectional study.<br>Sample size-245 males<br>Data collection- questionnaire.<br>Data analysis- statistical analysis.<br>Ethical approval- obtained.	50% had inadequate awareness of prostate cancer, 94.9% of the participants had knowledge of the condition and 54.1% aware of screening.<br>35.1% identified family history as risk factor.<br>Knowledge about screening services was low, only a quarter having prior knowledge about prostate specific antigen testing.<br>84.9% of respondents had positive attitudes towards prostate cancer.<br>Low awareness and access to screening services may contribute to poor attendance rates	To educate males about the risk factors, symptoms, diagnosis, and treatment through community-based programmes to increase awareness and uptake of screening.	The sample size used for the study was over estimated leading to high odds ratio.

[57] Morrison et al. (2017) Prostate cancer knowledge, prevention, and screening behaviours in Jamaican men.<br><br>Jamaican.	To establish the knowledge and attitudes facing screening and risks and prevention for prostate cancer.	Quantitative cross-sectional study.<br>Sample size-55 men.<br>Data collection-questionnaire.<br>Data analysis- statistical analysis.<br>Ethical approval- obtained.	Age, family history, and black race are the three non-modifiable risk factors for prostate cancer.<br>Diet and exercise level are some of the modifiable risk factors of prostate cancer.<br>Prostate specific antigen and digital rectal examination appropriate tool for prostate cancer diagnosis.<br>80% had positive behaviours towards prostate cancer screening and prevention.	Future strategies should focus on improving knowledge for prostate cancer and exercise among black population in Jamaica.	Sampling and selection bias with small sample size.
[98] Nijs et al. (2000). Why do men refuse or attend population-based screening for prostate cancer.<br><br>United States of America.	To examine, in connection to various background variables, the motivations for attending or declining population-based prostate cancer screening.	Qualitative cross-sectional study.<br>Sample size- 1,126 (500 refusers and 626 consecutive attenders).<br>Data collection-questionnaire.<br>Data analysis- statistical analysis.<br>Ethical approval- obtained.	Refusing screening is driven by discomfort.<br>Refusers are older, unmarried, and have lower education.<br>Screening uptake declined 42% in recent years; improvements include reminders, non-response reply chart, and adjusted uptake rates for men.	The study recommends considering health promotional aspects, particularly urological complaints, in designing strategies for reaching the general population.<br>Further research should explore underlying motives for refusal using qualitative and quantitative methods, considering pros and cons.	Self-reported study.<br>Low response rate.

[84] Ogunsanya et al. (2017) Knowledge for prostate cancer and screening among young multi-ethnic black men.<br><br>United States of America.	To define knowledge for prostate cancer and screening among young black men and also identify factors associated with prostate cancer knowledge.	Qualitative cross-sectional study.<br>Sample size- 267 black men.<br>Data collection- questionnaire.<br>Data analysis- statistical analysis.<br>Ethical approval- obtained.	Screening has been recognised as the best way to identify prostate cancer.<br>Most black men face several obstacles such as lack of knowledge, racism, and prostate cancer disparity gaps.<br>77.5% had never been screened for prostate cancer and some had a negative health screening experience.	Future studies should be considered to include older Black men (of screening age) to compare such differences in knowledge scores and its correlates.	Deficit in sampling several obstacles faced by participants.<br>Random answer due to lack of understanding of question asked.
[58] Pernar et al. (2018) The epidemiology for prostate cancer.<br><br>United States of America.	To discuss the state of evidence for specific genetic, lifestyle, and dietary factors in relation to prostate cancer risk.	Discussion paper.	The use of prostate specific antigen screening for prostate cancer is complex.<br>Risk factors such as age, race/ethnicity, family history, and genetic variants were primarily considered as non-modifiable.	Further research is needed to better understand disease heterogeneity molecular characterization for prostate cancer tumours.<br>Further research is needed to increase and integrate knowledge and develop targeted interventions that make best use of epidemiological interventions for prostate cancer patients.	Opinion paper only non-research based.
[59] Randazzo et al. (2016) A positive family history as a risk factor for prostate cancer in a population-based study with organised prostate-specific antigen screening: results of the Swiss European Randomised Study of	To evaluate the importance of a positive family history as a risk factor for prostate cancer incidence and grade in men undertaking a population-based trial of prostate-specific antigen screening.	Quantitative cross-sectional study.<br>Sample size- 5,129 men.<br>Data collection- questionnaire.<br>Data analysis – statistical analysis.<br>Ethical approval– not stated.	Men with a positive family history have higher prostate cancer diagnosis risk compared men with a negative family history, with comparable detection rate but weaker predictive effect.	To perform a more customized prostate cancer screening, information on positive family history, including detailed information on cancer features of the relative, may be required.	Incomplete data on the evaluation of family history and potential changes in family history data over time.

Screening for Prostate Cancer (ERSPC, Aarau).<br><br>Switzerland.			Opportunistic prostate specific antigen screening increases prostate cancer incidence, but overdiagnosis rate is 50%, and true hereditary cancers have shorter lead times and increased aggressive disease risk.		
[81] Riviere et al. (2023) Patient perspectives of prostate cancer screening vary by race following 2018 guideline changes.<br><br>United States of America.	To explore patient perspectives on prostate specific antigen screening, including perception rates, physician conversations, and shared decision-making.	Quantitative cross-sectional study.<br>Sample size- 43,685 men (40,301 non-Hispanic white men and 3384 African American).<br>Data collection- questionnaire.<br>Data analysis– statistical analysis.<br>Ethical approval– not stated.	African American men underrepresented in prostate cancer screening due to income, and education.<br>Access to care, primary care and health insurance had minimal impact on African American men lower prostate specific antigen screening rates.<br>The lower prostate specific antigen screening rate in African American men is likely due to systemic bias and medical mistrust, which contribute to decreased shared decision-making.	Future research should investigate systemic biases, self-advocacy and trust in the medical system.	Non-validated questionnaire.<br>Low representation of African American men.
[60] Sasagawa and Nakada (2001) Epidemiology of prostatic cancer in East Asia.<br><br>Japan.	To highlight the changes in trends and risk facts for prostate cancer over time.	Expert opinion paper	Prostate cancer has surged among the Asian community and risk factors such as diet, genetics, family history, race and age.<br>No evidence to support hormones causing prostate cancer.	Future research should focus on the epidemiological factors that may be useful for the prevention of prostate.	Opinion paper non research based.

[61] Schreiber-Agus (2001) Prostate cancer, basic characteristics and experimental models.<br><br>United States of America.	To discuss the basic and experimental models for prostate cancer.	Discussion paper.	Aetiology of prostate cancer is speculative. However, age, race, chemical exposure and family history are some predisposing factors. Prostatic specific antigen test and digital rectal examination most effective way to screen for prostate cancer. Prostate biopsy generally indicative of prognosis as helps determine type of treatment to be administered.	Future research is needed to improve on the pharmacological and alternative treatments for prostate cancer.	Opinion paper non-research based.
[69] Syrigos et al. (2005) Prostate cancer in the elderly.<br><br>United Kingdom.	To discuss existing evidence on screening, diagnosis and treatment for prostate cancer in the elderly.	Expert opinion.	Age is seen a major predisposing factor for prostate cancer. Prostate specific antigen testing, transrectal ultrasound and guided biopsy has shown to be effective tools in screening and diagnosing prostate cancer.	Further studies required to examine elderly patients' survival, preference, and quality of life in regard to prostate cancer.	Expert opinion paper non-research based.
[95] Taneja (2004) Imaging in the diagnosis and management for prostate cancer.<br><br>United States of America.	To discuss imaging techniques in the diagnosing and managing prostate cancer.	Expert opinion.	Prostate specific antigen test and digital rectal exam mostly used for prostate specific antigen screening. Transrectal ultrasound is also used in corelation with prostate biopsy to confirm diagnosis. Clinicians rely on endorectal coil magnetic resonance imaging, magnetic resonance	Future research should focus on the role for imaging in the management of prostate cancer.	Expert opinion paper non-research based.

			spectroscopic imaging for diagnosing prostate cancer		
[62] Terries et al. (2005) Primary care providers' attitudes toward prostate cancer risk, factors at a veteran's affairs health care facility.<br><br>United States of America.	To explore factors that influence primary care providers' decision to screen patients for prostate cancer	Quantitative cross-section study.<br>Sample size- 175 (102 physicians and 73 nurses' practitioners).<br>Data collection- questionnaire.<br>Data analysis – thematic analysis<br>Ethical approval– not stated.	African American race or having a family history for prostate cancer were the two main factors that increased the likelihood for screening.<br>A history of smoking and/or vasectomy prompted screening.<br>As population, veterans with a history of Agent Orange exposure were a significantly factor in screening.<br>The individual's own anxiety regarding prostate cancer increased the tendency for screening.	Education and awareness needed to improve.<br>Physicians should describe the potential benefits and known harms of screening, diagnosis, treatment and listen patient's concerns and then individualize the decision to screen.<br>Men 50+ and men 40+ with a family history should have an annual prostate examination (digital rectal examination and prostate specific antigen test).	Non validated questionnaire may affect generalizability.
[73] Trudeau (2020) Dietary patterns are associated with risk of prostate cancer in a population-based case-control study in Montreal, Canada.<br><br>Canada.	This study shows the relationship between food patterns and prostate cancer risk in a population-based case-control study conducted in Montreal, Canada (2005-2012).	Quantitative cross-section study.<br>Sample size- 1,919 cases (men adhering to healthy eating).<br>Data collection- questionnaire.<br>Data analysis– statistical analysis.<br>Ethical approval– obtained	Men with healthy eating patterns have lower overall and high-grade prostate cancer risk, while Western sweet and beverages patterns of some men increase risks of getting prostate cancer.	The study identified novel dietary patterns and hypothesis-generating associations, requiring further confirmation in future studies.	Non validated questionnaire may affect generalizability.
[63] Turner and Drudge-Coates (2010) Prostate cancer: risk factors, diagnosis and management.	To discuss prostate cancer in relation to its incident, epidemiology, risk factors, symptoms and treatment.	Discussion paper.	Cause of prostate cancer may not be known. However, age, family history, race, geographical location are predisposing factors.	Nurses need to understand the risk factors and screening options to help them provide safe and quality care.	Expert opinion paper non-research based.

United States of America.			Conversely, the progress of latent to clinical prostate cancer may be caused by high fat diet, sexual behaviour, alcohol consumption and occupational exposure.<br>To screen and diagnose a physical examination digital rectal examination, prostate specific antigen test and prostate biopsy is needed.		
[64] Vane (2019) Prostate Cancer Screening: A Review of Current Recommendations.<br><br>United States of America.	To discuss prostate cancer screening barriers in the clinical setting and outline some current recommendation.	Discussion paper.	Risk for prostate cancer grouped into two types, modifiable risk factor (consumption of alcohol, smoking, sedentary lifestyle and diet) and non-modifiable risk factor (age, ethnicity, family history, genetics, and geographical location).<br>The use of prostate specific antigen, digital rectal examination and prostate biopsy has improved in the early detection and screening of prostate cancer. However, it has led to overdiagnosis and overtreatment.	Surveillance and education of patients on modifiable risk factor will help reduce risk factors for prostate cancer.<br>There is the need to discuss screening option with patient before health provide decide on the type of screening option to use.	Expert opinion paper non-research based.
[65] Watson and Austoker (2008) Testing for prostate cancer.<br><br>United Kingdom.	To discuss how prostate cancer is tested.	Discussion paper.	Main cause for prostate cancer is unknown. Factors such as age, race, hereditary has been recognized as common	There should be continuous education, counselling and communication between the patient and the nurse	Expert opinion paper non-research based.

			risk factors for prostate cancer.<br>In terms screening digital rectal examination and prostate specific antigen test are the most endorsed and if any uncertainty prostate biopsy is used to confirm the diagnosis.	before prostate specific antigen screening to help reduce fears and anxiety.	
